# Design Principles and Applications of Selective Lanthanide-Based Receptors for Inorganic Phosphate

**DOI:** 10.3389/fchem.2022.821020

**Published:** 2022-02-07

**Authors:** Valérie C. Pierre, Randall K. Wilharm

**Affiliations:** Department of Chemistry, University of Minnesota, Minneapolis, MN, United States

**Keywords:** phosphate, receptor, lanthanide, luminescence, supramolecular, recognition

## Abstract

Phosphate is an anion of both environmental and medical significance. The increase in phosphate levels in surface waters due primarily to run-offs from fertilized agricultural fields causes widespread eutrophication and increasingly large dead-zones. Hyperphosphatemia, a condition in which blood phosphate levels are elevated, is a primary cause of increased mortality and morbidity in chronic or advanced kidney disease. Resolving both of these issues require, in part, new technology that could selectively sequester phosphate in water at neutral pH. The high hydration energy of phosphate, which prevents organic receptors from functioning in water with sufficient affinity, can be overcome via coordination to a hard metal ion. The hardness, oxophilicity and lability of lanthanide ions make them excellent candidates for the design of high affinity phosphate receptors. In this perspective, we discuss how the principles of lanthanide coordination chemistry can be exploited to design sensitive and selective receptors for phosphate. Unlike many supramolecular systems, these hosts do not recognize their anionic guests via directed electrostatic and hydrogen bonding interactions. Instead, the selectivity of our fluxional receptors is governed entirely by acid-base chemistry and electrostatic forces. Parameters that affect the affinity and selectivity of the receptors include the basicities of the coordinating ligand and of the targeted anion, the acidity of the lanthanide ion, and the geometry of the ligand. Uniquely, their affinity for phosphate can be readily tuned by orders of magnitude either by peripheral interactions or by the lanthanide ion itself without affecting their exquisite selectivity over competing anions such as bicarbonate and chloride.

## Introduction–Need and Requirements for Phosphate Receptors

Phosphate, a tetrahedral oxyanion with one of the largest hydration energy (ΔG_hydration_ H_2_PO_4_^−^ = -465 kJ mol^−1^), (Marcus, 1991), has received much attention in supramolecular chemistry due to its significance in both environmental and medical applications. Environmentally, phosphate is the leading cause of eutrophication due to agricultural run-offs into lakes, rivers and coastal waters. (Schindler et al., 2008; Wang and Wang, 2009; Ménesguen et al., 2018). Efforts to clean up or attenuate these damages would benefit from the development of novel technology that can selectively remove and recover phosphate from wastewaters. Medically, the poor ability of dialysis to remove the highly hydrated anion leads to accumulation of phosphate in patients with advanced or chronic kidney diseases. (Sherman, 2016). With time, this accumulation leads to vascular calcification with significant increased risk of mortality and morbidity. (Block et al., 1998; Ganesh et al., 2001; Block et al., 2004). Phosphate binders such as lanthanum carbonate, which are taken orally at large doses, lead to significant side effects and have shown limited efficacy at managing this conditions. (Floege, 2016; Biruete et al., 2020).

Both of these problems could be addressed with efficacious supramolecular receptors that could sequester excess phosphate from complex aqueous media. There are similarities in the requirements for both of these applications, including efficacy in water at neutral pH and extremely high selectivity over other anions present in higher concentration, notably bicarbonate and chloride. There are also differences. Because the target phosphate level in blood is 1.5 mM, phosphate receptors with relatively low affinity will suffice as long as they are highly selective and highly stable. For environmental applications, prevention of eutrophication and algae blooms requires decreasing phosphate levels to below 1 µM, (Great Lakes Water Quality Agreement, 2012), requiring receptors with substantially higher affinity for the anion.

Many receptors for phosphate have been reported and reviewed. (Hargrove et al., 2011; Pal et al., 2020; Ramakrishnam Raju et al., 2020). Most are organic molecules inspired by phosphate binding proteins and follow the traditional lock-and-key principles of supramolecular chemistry. In these cases, phosphate recognition is contingent on pre-organized hydrogen-bonding and electrostatic interactions, which are too weak to overcome the very high hydration energy of phosphate. As a result, they perform poorly or not at all in aqueous media. Overcoming the hydration energy of phosphate, a hard anion, is best achieved by enabling the anion to coordinate a hard metal. Some success has been achieved with zinc(II) and copper (II), although the affinity and selectivity of these receptors remain insufficient for the intended medical and environmental applications. Lanthanide complexes, on the other hand, offer the possibility to achieve not only very high selectivity and sensitivity, as required for both of these applications, but also the ability to tune the affinity for phosphate as desired. Herein we discuss how and why our group has achieved this unique control.

## Lanthanide-Based Receptors for Phosphate

The affinity of lanthanide complexes with one or more open coordination sites for inorganic phosphate in water is well known. All clinical gadolinium-based magnetic resonance imaging (MRI) contrast agents have at least some affinity for phosphate. (Burai et al., 1997). Many lanthanide-based probes for smaller anions such as HCO_3_^−^ are not perfectly selective over phosphate despite their more crowded open coordination sites that disfavors coordination of the bulkier tetrahedral anion. (Bretonniere et al., 2004; Pierre et al., 2006a; Pálinkás et al., 2009; Smith et al., 2012). It thus follows from these observations that one could design lanthanide complexes that have good affinity for phosphate simply by opening up at least one coordination site. (Ramakrishnam Raju et al., 2020). Importantly, one should note that for lanthanide receptors in water and in the absence of anions, these open coordination sites are occupied by loosely bound and rapidly exchanging water molecules. Selectivity over other anions, most of which are smaller (i e., F^−^, HCO_3_^−^), is more difficult to engineer since it cannot be achieved by steric means. Instead, this selectivity that is so crucial to both medical and environmental applications has to be incorporated by modifying the nature of the coordinating ligand L.

**FIGURE 1:**
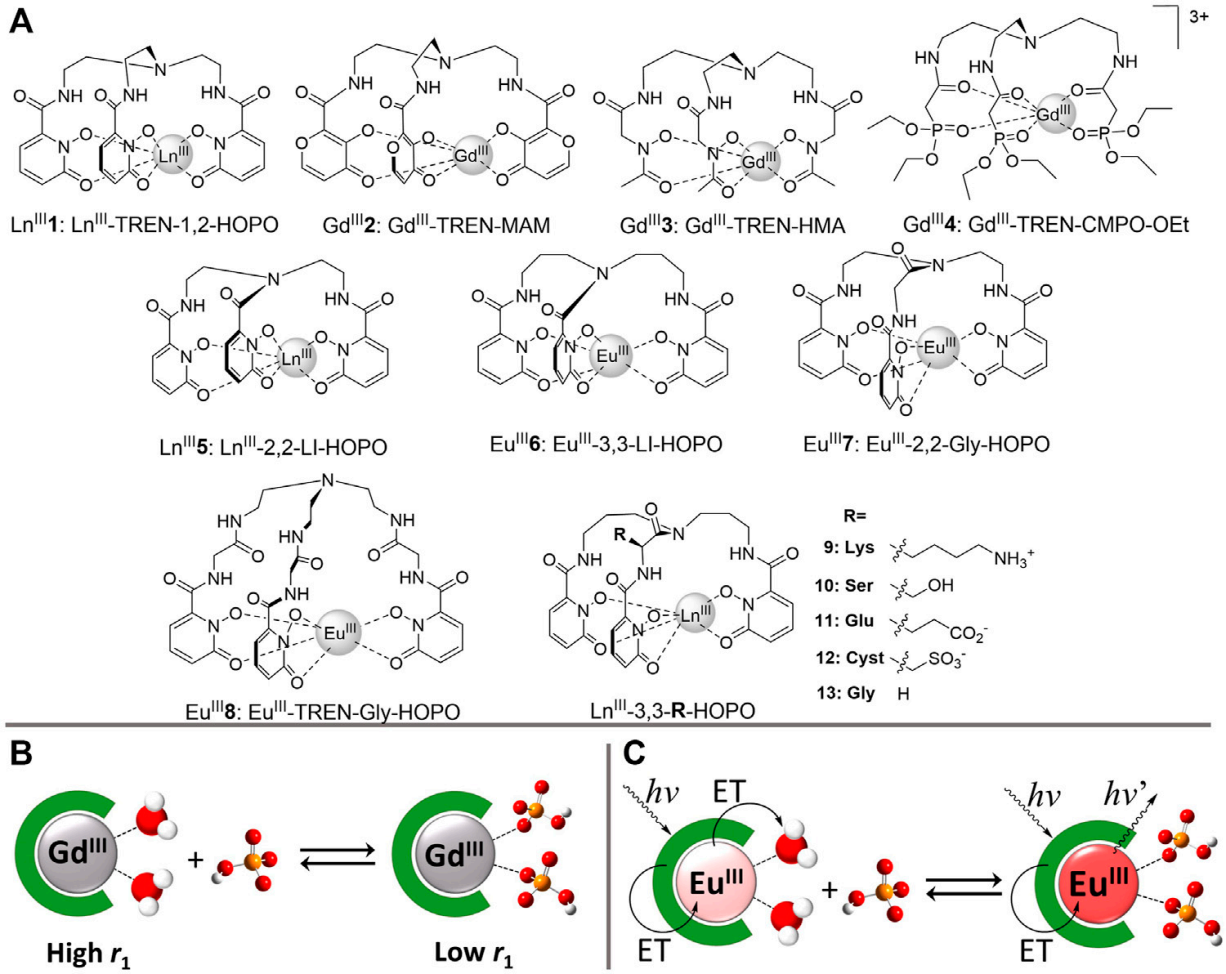
**(A)** Chemical structures of phosphate receptors **(B)** Relaxivity response of Gd^III^ receptors to phosphate binding. Rapidly exchanging water molecules bound to Gd^III^ enhance the proton relaxation of the bulk water, resulting in high longitudinal relaxivity (*r*_1_). Bound phosphate anions prevent coordination of water molecules, thereby reducing the proton relaxation of the bulk solvent and decreasing the relaxivity (*r*_1_) **(C)** Luminescence response of Eu^III^ receptors to phosphate binding. Coordinated water molecules efficiently quench Eu^III^ luminescence. Phosphate anions displace the inner-sphere water molecules upon coordination, which increases the Eu^III^-centered luminescence intensity and lifetime.

Insight into how selectivity can be engineered also comes from the field of MRI contrast agents and luminescent probes. Europium(III) complexes of TREN-1,2-hydroxypyridinonate (**1,** Figure 1A, Table 1), are known to have no affinity for phosphate despite their flexible coordination geometry that leaves two sterically unhindered open coordination sites. (Jocher et al., 2007). They thus differ from their less basic polyaminocarboxylate counterparts that also bind oxyanions such as phosphate and bicarbonate. This difference is rational. Since the filled 5s and 5p orbitals efficiently shield the partially filled 4f orbitals of the lanthanides, bonding to lanthanide ions is almost entirely ionic. Unlike transition metal complexes, there is negligible overlap between the molecular orbitals of the lanthanide ion and those of the coordinating ligands. Coordination to lanthanide ions is thus primarily an acid-base interaction. There is thus, predictably, a well-established relationship between the basicity of the coordinating ligand L and the stability of Gd^III^L complexes. The more basic the ligand, the more stable the complex, up to a point near Σp*K*_a_ ≈ 35 beyond which the trend reverses. (Kumar et al., 1993; Doble et al., 2003; Pierre et al., 2004).

**TABLE 1:** Inorganic phosphate ternary complex stoichiometry, affinity, selectivity, and mode of detection for phosphate receptors.

Receptor	Stoichiometry (LnL:Pi)	Affinity (log*β*)^a^	Interfering anions (log*β*)^a^	Mode of detection	Ref
Eu^III^ **1**	No binding	**—**	**—**	luminescence	20
Gd^III^ **1**				relaxivity	24
Gd^III^ **2**	1:2	17	arsenate (7.8)	relaxivity	24
			bicarbonate (2.7)		
			fluoride (2.0)		
Gd^III^ **3**	transmetallation	6.1	arsenate (11)	relaxivity	24
			bicarbonate (3.0)		
			fluoride (2.6)		
Gd^III^ **4**	transmetallation	29	arsenate (4.7)	relaxivity	24
			bicarbonate (3.0)		
			fluoride (3.1)		
Sm^III^ **5**	1:2	10.4	not tested	luminescence	35
Eu^III^ **5**	1:2	10.4	none	luminescence	29
Gd^III^ **5**	1:2	12.6	not tested	luminescence	35
Eu^III^ **6**	No binding	**—**	**—**	luminescence	29
Eu^III^ **7**	No binding	**—**	**—**	luminescence	29
Eu^III^ **8**	No binding	**—**	**—**	luminescence	29
Eu^III^ **9**	1:2	11.3	none	luminescence	38
Eu^III^ **10**	1:3	15.9	none	luminescence	38
Eu^III^ **11**	No binding	**—**	**—**	luminescence	38
Eu^III^ **12**	No binding	**—**	**—**	luminescence	38
Sm^III^ **13**	1:3	14.7	not tested	luminescence	35
Eu^III^ **13**	1:3	14.5	none	luminescence	29
Gd^III^ **13**	1:3	17.0	not tested	luminescence	35

a Overall association constant for the indicated anion, which corresponds to the equilibrium Ln^III^L + *n*A^−^ ⇋ Ln^III^L∙A_*n*_ where *β* = [Ln^III^L∙A_*n*_]/([Ln^III^L][A^−^]^*n*^), A^−^ is the indicated anion and *n* is the total number of anions found to bind to the receptor.

We postulated that this same trend could be used to rationally design selective lanthanide receptors for phosphate. Since phosphate is not just the guest of the lanthanide host but also one of its ligands, it would thus follow that the basicity of the ligand L should impact the affinity of the Gd^III^L complex for phosphate and other anions. TREN-1,2-hydroxypyridinonate (**1**) ligands, which have a Σp*K*_a_ ≈ 35, already fulfill the needs of the gadolinium(III) ion. Coordination of a basic oxyanion guest would increase the sum of the basicity for all coordinating ligands, which is thermodynamically unfavorable. The opposite is true for complexes that use less basic ligands. For those, coordination of an additional oxyanion would be thermodynamically favored because it would increase the Σp*K*_a_.

Indeed, we have demonstrated that if the geometry of the ligand and the charge of the complex are constant, and in the absence of steric hindrance at the open coordination sites, the affinity of gadolinium(III) complexes for anions is directly determined by only two factors: the stability of the complex, which is itself a function of the basicity of the ligand, and the basicity of the anionic guest. (Ramakrishnam Raju et al., 2019). The more stable the Gd^III^L complex, the lower the affinity of the lanthanide for the anion. The most stable Gd^III^ complex has no affinity for any anions (Table 1). The lanthanide complexes with intermediate stability form ternary (Ln^III^L·anion) or quaternary (Ln^III^L·anion_2_) assemblies with anions. For weakly stable complexes, however, phosphate and other anions readily compete with and displace the ligands L, resulting in the formation of Ln^III^·anion_x_ precipitates. Advantageously, these equilibriums can be readily monitored by relaxivity. Since the relaxivity (*r*_1_) of Gd^III^L complexes is directly proportional to the number of inner-sphere water molecules (Figure 1B), unbound Gd^III^L receptors have significantly higher *r*_1_ than those for which the inner-sphere water molecules have been displaced by coordinating anions.

The reverse—selectivity between different anions for a given lanthanide complex—follows the same rules. In the absence of steric hindrance at the coordination site, the affinity of Gd^III^ complexes for anions always follows the trend in the basicity of the anions: phosphate > arsenate > bicarbonate > fluoride. (Ramakrishnam Raju et al., 2019). That basicity, and not Pearson’s hardness, governs the selectivity between anions is due to the purely ionic nature of lanthanide coordination. Indeed, under basic conditions, significantly softer but also more basic anions such as CN^−^ also coordinate lanthanide ions. (Huang and Pierre, 2018).

The ligand TREN-MAM (**2**) is slightly less basic than TREN-HOPO (**1**). (Puerta et al., 2006). As a result, even though Gd^III^-TREN-HOPO has no affinity for anions, Gd^III^-TREN-MAM has high affinity for phosphate and good, although not sufficient, selectivity over bicarbonate and fluoride. (Harris et al., 2017; Table 1). Predictably, given the role of basicity on anion coordination, this coordination is pH dependent. Gd^III^-TREN-MAM binds phosphate with high affinity at neutral pH but not at all at pH 2. This behavior, which is common to the entire class of lanthanide-based phosphate receptors, is key to pH-driven catch-and-release process that, advantageously, enable recycling of the receptor over numerous cycles. (Harris et al., 2017).

It is important to note that the above trends are only valid in so much as there is no significant steric hindrance at the open coordination sites. This is not the case, for instance, for macrocyclic polyaminocarboxylate (DOTA-type) complexes. Crystal structures of lanthanide complexes of the class of tris-bidentate ligands indicate that their open coordination sites, filled with solvent molecules in the absence of anions, are far apart from each other. (Xu et al., 2004; Jocher et al., 2007). There is no steric hindrance preventing anion coordination. Moreover, in the absence of ligand-field stabilization and with non-rigid ligands such as the ones discussed herein, lanthanide complexes are highly fluxional, interconverting rapidly between different isomers close in energy. This rapid interconversion between numerous isomers, which is faster than the NMR time-scale, is apparent for both the unbound (free) and the phosphate-bound form of the complexes. It is evident that this class of fluxional lanthanide hosts does not follow the standard lock-and-key geometric standards of supramolecular chemistry articulated by Emil Fischer whereby recognition of the phosphate guest is reliant on pre-organized hydrogen-bonding and electrostatic interactions. Instead, the affinity and selectivity for phosphate is entirely governed by the highly ionic lanthanide(III)-OPO_3_H bond.

Gd^III^-TREN-MAM demonstrated the potential to rebrand failed gadolinium-based MRI contrast agents as phosphate sequestration agents, but its insufficient selectivity over bicarbonate does not make it the receptor of choice for both medical and environmental applications. Based on the above discussion, a complex of stability intermediate between TREN-MAM and TREN-HOPO should offer the necessary selectivity. Given the limited choice in podands, a simpler approach to achieve this goal than changing the podand is to slightly destabilize the Gd^III^-TREN-HOPO complex by marginally modifying its geometry. With that goal in mind, we synthesized five more tripodal Eu^III^-HOPO complexes bearing different ligand caps (Figure 1A). (Huang et al., 2020) Europium(III) was chosen for this study instead of gadolinium(III) due to the lower solubility of the lanthanide HOPO complexes that is incompatible with the insensitive nature of relaxometric measurements. The high efficiency by which HOPO sensitizes Eu^III^ emission, on the other hand, enables facile determination of anion coordination by time-resolved luminescence spectroscopy (Figure 1C).

Of the six Eu^III^-HOPO complexes synthesized, four have eight-coordinate, q = 2 ground states (Eu^III^ **1**, Eu^III^ **5**, Eu^III^ **6**, and Eu^III^ **7**), whereas two have nine-coordinate, q = 3 ground states (Eu^III^ **8** and Eu^III^ **13**), where q is the number of open coordination sites initially filled by water molecules. This observation is in line with prior reports that demonstrated that the 8 and 9 coordination states of the HOPO-class of complexes are very close in energy such that minor modification in ligand geometry is sufficient to favor one or the other state. (Pierre et al., 2006b). Two of these complexes: the 8-coordinate Eu^III^-2,2-LI-HOPO (Eu^III^ **5**) and the 9-coordinate Eu^III^-3,3-Gly-HOPO (Eu^III^ **13**) bind phosphate with very high affinity in water at neutral pH. Predictably, the Eu^III^ complex with two open coordination sites binds two phosphate anions, thereby forming a Ln^III^L·Pi2 assembly, whereas the one with three open coordination sites binds three phosphate anions, forming Ln^III^L·Pi3. Importantly, both complexes showed not only high affinity for phosphate, but also exquisite selectivity over other anions, notably bicarbonate, fluoride and carboxylates. As such, they both fulfill all the prerequisites noted above for both environmental and medical applications of phosphate sequestration.

This study yielded two important conclusions. The first is that there is no relationship between the number of open coordination sites and the affinity of the complex for anions. Some highly stable complexes with three open coordination sites have no affinity for phosphate or any other anions. Some slightly less stable complexes with only two open coordination sites do have high affinity for phosphate. The second is that there is, however, a strong relationship between the quantum yields of europium complexes of 1,2-HOPO and their affinity for phosphate. Indeed, the only two complexes that bind phosphate have noticeably lower quantum yields. Raymond previously reported that lower quantum yields of some 1,2-HOPO Eu^III^ complexes are due in part to the effect to the increased distance separating the HOPO sensitizer from the Eu^III^ ion. (Abergel et al., 2009; Daumann et al., 2016). Decreasing the effectiveness by which one or more 1,2-HOPO podand coordinates Eu^III^ is what leads to increase affinity of the receptor for phosphate. Unfortunately, it is not yet possible to predict or compute which ligand will or will not destabilize the complex, that is, which ligand cap will decrease the effectiveness by which 1,2-HOPO coordinates Eu^III^ as necessary to enable phosphate coordination.

Altogether, these studies demonstrate that highly sensitive and selective receptor for phosphate that function in water at neutral pH can be successfully designed by optimizing the stability of the lanthanide coordination sphere. A common theme in achieving our goal was the importance of the basicity of both the coordinating ligand L and the anions. It thus follows that the nature of the lanthanide ion, which affects its Lewis acidity, should also have an effect on the stability of the complex and, by extension, on the affinity of the receptor for phosphate.

The most important periodic trend across the lanthanide series is their gradual contraction, with a 16% reduction in the ionic radii of the lanthanides from La^III^ to Lu^III^. (Shannon, 1976). This contraction results in a progressive increase in the acidity of the lanthanides across the series from a p*K*_a_ of 9.24 for La^III^ to 7.52 for Lu^III^. (Klungness and Byrne, 2000). Our NMR studies demonstrated that these tris-bidentate ligands are highly fluxional suggesting that steric hindrance does not impede lanthanide coordination. Given that the stability of gadolinium(III) complexes is a function of the basicity of the ligand, we anticipated a linear progression in stability of Ln^III^-2,2-LI-HOPO complexes across the series. Indeed, we observed significant and progressive increase in pM values from La^III^ to Lu^III^ indicating that for complexes of this class of ligand, steric components do not hinder coordination of even the smallest lanthanides. (Wilharm et al., 2021). The extreme difference in pM values between Lu^III^ and La^III^, ΔpM = 7.5, which is substantially more than that observed with other ligands, suggest that this class of ligands also holds potential for separation of rare earth elements.

The same periodic trend is observed for the affinity of lanthanide complexes for phosphate. The affinity for phosphate of both Ln^III^-2,2-LI-HOPO and Ln^III^-3,3-Gly-HOPO increases significantly from La^III^ to Lu^III^, with a difference in affinity (Δlog*β*) for the oxyanion of at least 5.2 and 2.5 between the first and last lanthanide complexes of those two ligands, respectively. (Wilharm et al., 2021). As predicted, the affinity of the lanthanide receptors for phosphate can be tuned as desired by orders of magnitude simply by moving left or right on the periodic table. Advantageously, increased efficacy does not come at the detriment of complex stability. lutetium(III) has the highest affinity for phosphate and also forms the most stable 1,2-HOPO complexes. It thus holds particular potential for translational applications for both medical and environmental applications since it is even less likely, given its stability, to leach from the receptor.

The unique ability to design lanthanide complexes that bind phosphate with high affinity and selectivity stems from the purely ionic nature of the lanthanide-ligand interactions. By extension, this ionic nature of lanthanide coordination stipulates that, even in water, peripheral electrostatic charges would have a significant effect on the affinity of the receptor for the oxyanion. We previously exploited this behavior to distinguish between ATP, ADP and AMP and monitor enzymatic reactions of kinases. (Weitz et al., 2012; Weitz et al., 2013). The extreme magnitude of this effect was demonstrated with a series of five amino-acid derivatives, Eu^III^-3,3-AA-HOPO (Eu^III^ **9**–Eu^III^ **13**), that differed in the incorporation of a single positively charged (lysine), negatively charged (glutamate and cysteate) or neutral (serine) pendant group capable of hydrogen bonding (Figure 1A and Table 1). (Huang et al., 2019) In the absence of phosphate, all five of these complexes are nine-coordinate with three inner-sphere water molecules. The two negatively-charged complexes, Eu^III^-3,3-Glu-HOPO (Eu^III^ **11**) and Eu^III^-3,3-Cyst-HOPO (Eu^III^ **12**) have no affinity whatsoever for phosphate at neutral pH: addition of 10 equivalents of the anion does not increase either the luminescence intensity nor the luminescence lifetime of the probe. Addition of a single alcohol group that can hydrogen bond to phosphate and stabilize its coordination to the lanthanide center increases the affinity of the complex for the oxyanion by 20-fold. Whereas the two neutral complexes Eu^III^-3,3-Gly-HOPO (Eu^III^ **13**) and Eu^III^-3,3-Ser-HOPO (Eu^III^ **10**) each bind three phosphates, one for each open coordination site, the positively charged complex Eu^III^-3,3-Lys-HOPO (Eu^III^ **9**) only binds two. The pendant primary amine of the lysine derivative strongly stabilizes one of the inner-sphere water molecules. As a result, the Eu^III^-3,3-Lys-HOPO·Pi_2_ adduct retains one inner-sphere water molecule even in the presence of an excess of phosphate. Similar behavior has been observed with other gadolinium(III) 1-Me-3,2-HOPO complexes. (Pierre et al., 2006b). Nonetheless, even though Eu^III^-3,3-Lys-HOPO only binds two phosphates, which accounts for its lower increase in luminescence intensity and luminescence lifetime upon anion coordination, both the first and second association constant for phosphate increases over two orders of magnitude compared to the parent neutral glycine derivative. One can thus dramatically increase the affinity of a lanthanide receptor for phosphate by orders of magnitude by adding either peripheral positive charges or stabilizing hydrogen-bonding moieties. The presence of a single negative charge, on the other hand, is sufficient to eliminate all affinity for phosphate. Significantly, increasing or decreasing the affinity via peripheral interactions does not affect the exquisite selectivity of these receptors.

## Conclusion and Outlook

The above discussion highlighted the principles behind the design of lanthanide-based receptors with high affinity for phosphate and high selectivity over other common competing anions in water at neutral pH, notably bicarbonate. The high selectivity of these receptors is notable given the absence of steric hindrance at the open coordination sites and the lack of pre-organized hydrogen-bonding or electrostatic interactions. In these aspects, lanthanide-based receptors distinguish themselves from the majority of supramolecular hosts for anion recognition. The high stability of the complexes ensures that the metal will likely not leach into either a biological or environmental media, as prerequisite for any phosphate sequestration technologies. Future work will focus on immobilizing these receptors on polymers or materials and testing these unique filtration technologies in application such as the treatment of hyperphosphatemia and phosphate sequestration from wastewaters.
